# Publisher’s Notice

**Published:** 1868-12-01

**Authors:** 


### *EDITORIAL.*

### *Publisher's Notice.*

It is peculiarly gratifying to the conductors of a publication, with many weights and responsibilities to sustain, that the outside reader can know nothing of, to receive assurances of favor and esteem from his patrons. From the time of its commencement up to the beginning of the present year, THE JOURNAL was conducted by professional men whose one aim was to supply the want for such a periodical which the rapid growth of the profession had created. The attention of these was entirely absorbed in furnishing the most valuable articles and intelligence; so that it naturally happened that the business of the periodical, which is no less important and necessary to its existence than the editorial management, fell into neglect. This will account for the mistakes which required no inconsiderable amount of labor and trouble on the part of the present conductors to correct. Efforts have been made during the present year to secure the payment of the subscriptions on THE JOURNAL, many of which had been accumulating, and which amounted in the aggregate to a large sum. When, therefore, the bills were presented to the delinquent subscribers, some of them, having been long accustomed to receive THE JOURNAL for nothing, grumbled excessively, and request a second or third presentation of their account. The replies of these discontented spirits go to make up the sum of troubles which the editor is obliged to experience; but it was at least satisfactory to learn that the majority of them with the honorable spirit which ever characterizes the worthy members of the profession, did not repudiate so just a debt. It has been gratifying too, not a little, to receive remittances accompanied with an expression of appreciation of THE JOURNAL and of a determination to render prompt payment in future. The following characteristic letter was received while the last nnmber was in press:

*November* 10, 1868.

*To the Editor of* THE JOURNAL:

Your bill of November 1st is received. Enclosed find post-office order for $10, for which please receipt and return. I thank you for your indulgence. The times have been hard with me, or I should have remitted sooner. The offer of discount is a kind one upon your part, but I consider that THE JOURNAL is worth its price, and I send you the full amount. Continue sending it to me. I can't do without it. It is the best I have seen. Very respectfully.

Hereafter, all letters and communications to the Editor of THE JOURNAL will be addressed, CHICAGO MEDICAL JOURNAL, No. 71 Dearborn Street Chicago, Ill. The box at the post office has been discontinued.

